# Supplementary material for: Evidence-based hand hygiene: Liquid or gel handrub, does it matter?
Source: Antimicrob Resist Infect Control. 2023 Feb 13;12:12. doi: 10.1186/s13756-023-01212-4 (PMC9926746; doi:10.1186/s13756-023-01212-4)
Supplement: Supplementary file 1 — Additional file 1: Fig. S1. Liquid ABHR—in vitro evaporation rate examination results. Fig. S2. Gel ABHR—in vitro evaporation rate examination results. [file 13756_2023_1212_MOESM1_ESM.docx]

**Evidence-Based Hand Hygiene: Liquid or Gel Handrub, does it matter?**

**Constantinos Voniatis^1,2^**, Száva Bánsághi^3,6^, Dániel Sándor Veres^4^, Péter Szerémy^6^, Angela Jedlovszky-Hajdu^2^, Attila Szijártó^1^, Tamás Haidegger^6,7^

1. Department of Surgery, Transplantation and Gastroenterology, Semmelweis University, Budapest, Hungary
2. Laboratory of Nanochemistry, Department of Biophysics and Radiation Biology, Semmelweis University, Budapest, Hungary
3. Doctoral School of Health Sciences, Semmelweis University, Budapest, Hungary
4. Department of Biophysics and Radiation Biology, Semmelweis University, Budapest, Hungary
5. University Research and Innovation Centre (EKIK), Óbuda University, Budapest, Hungary
6. Austrian Center for Medical Innovation and Technology (ACMIT), Wiener Neustadt, Austria

Corresponding Author: Dr. Tamás Haidegger, [haidegger@irob.uni-obuda.hu](mailto:haidegger@irob.uni-obuda.hu)

**Supplementary Material**


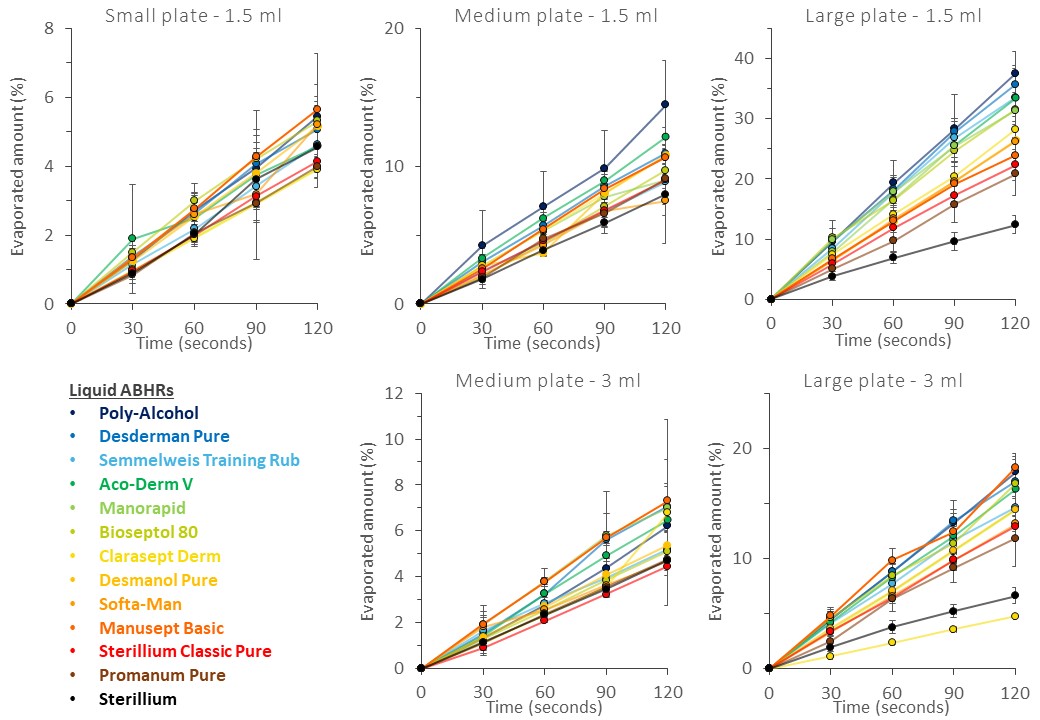


Supporting Figure 1. Liquid ABHR – in vitro evaporation rate examination results

**
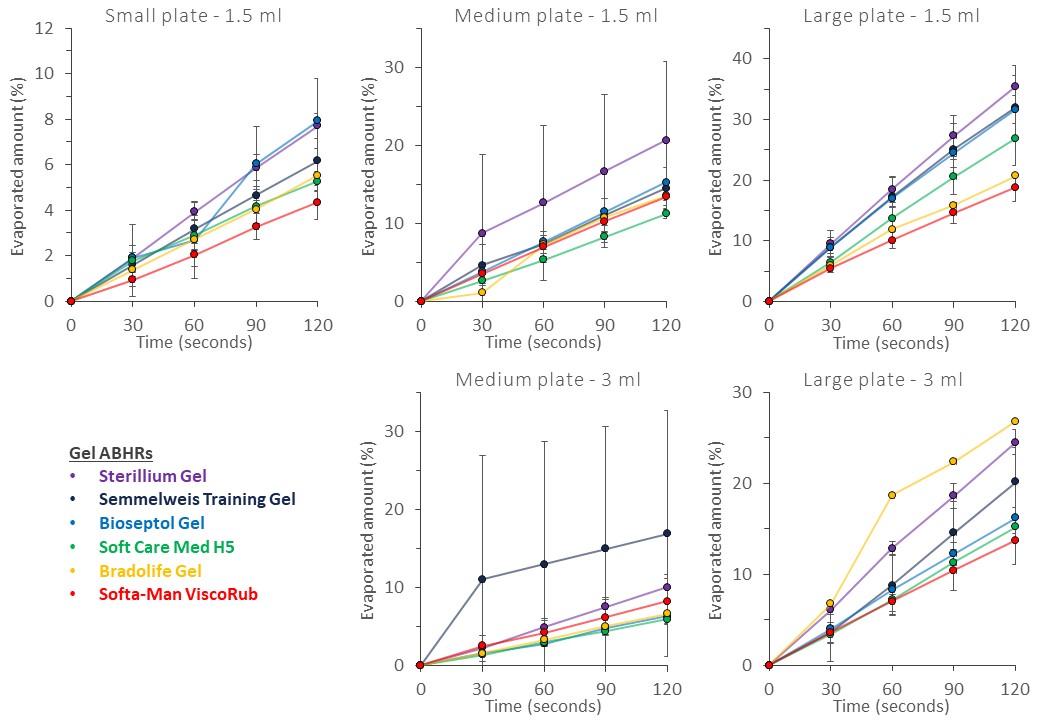
**

Supporting Figure 2. Gel ABHR – in vitro evaporation rate examination results
